# Identifying digital biomarkers of illness activity and treatment response in bipolar disorder with a novel wearable device (TIMEBASE): protocol for a pragmatic observational clinical study

**DOI:** 10.1192/bjo.2024.716

**Published:** 2024-08-01

**Authors:** Gerard Anmella, Filippo Corponi, Bryan M. Li, Ariadna Mas, Marina Garriga, Miriam Sanabra, Isabella Pacchiarotti, Marc Valentí, Iria Grande, Antoni Benabarre, Anna Giménez-Palomo, Isabel Agasi, Anna Bastidas, Myriam Cavero, Miquel Bioque, Clemente García-Rizo, Santiago Madero, Néstor Arbelo, Andrea Murru, Silvia Amoretti, Anabel Martínez-Aran, Victoria Ruiz, Yudit Rivas, Giovanna Fico, Michele De Prisco, Vincenzo Oliva, Aleix Solanes, Joaquim Radua, Ludovic Samalin, Allan H. Young, Antonio Vergari, Eduard Vieta, Diego Hidalgo-Mazzei

**Affiliations:** Digital Innovation Group, Bipolar and Depressive Disorders Unit, Institute of Neuroscience, Department of Psychiatry and Psychology, Hospital Clínic de Barcelona, Spain; Department of Psychiatry and Psychology, Institut d'Investigacions Biomèdiques August Pi i Sunyer (IDIBAPS), Barcelona, Spain; Biomedical Research Networking Centre Consortium on Mental Health (CIBERSAM), Instituto de Salud Carlos III, Madrid, Spain; Department of Medicine, School of Medicine and Health Sciences, University of Barcelona, Spain; and Institute of Neurosciences (UBNeuro), University of Barcelona, Spain; School of Informatics, University of Edinburgh, UK; Digital Innovation Group, Bipolar and Depressive Disorders Unit, Institute of Neuroscience, Department of Psychiatry and Psychology, Hospital Clínic de Barcelona, Spain; Digital Innovation Group, Bipolar and Depressive Disorders Unit, Institute of Neuroscience, Department of Psychiatry and Psychology, Hospital Clínic de Barcelona, Spain; Department of Medicine, School of Medicine and Health Sciences, University of Barcelona, Spain; and Institute of Neurosciences (UBNeuro), University of Barcelona, Spain; Digital Innovation Group, Bipolar and Depressive Disorders Unit, Institute of Neuroscience, Department of Psychiatry and Psychology, Hospital Clínic de Barcelona, Spain; Department of Psychiatry and Psychology, Institut d'Investigacions Biomèdiques August Pi i Sunyer (IDIBAPS), Barcelona, Spain; Biomedical Research Networking Centre Consortium on Mental Health (CIBERSAM), Instituto de Salud Carlos III, Madrid, Spain; Department of Medicine, School of Medicine and Health Sciences, University of Barcelona, Spain; Institute of Neurosciences (UBNeuro), University of Barcelona, Spain; and Barcelona Clinic Schizophrenia Unit, Institut d'Investigacions Biomèdiques August Pi i Sunyer (IDIBAPS), Barcelona, Spain; Digital Innovation Group, Bipolar and Depressive Disorders Unit, Institute of Neuroscience, Department of Psychiatry and Psychology, Hospital Clínic de Barcelona, Spain; Biomedical Research Networking Centre Consortium on Mental Health (CIBERSAM), Instituto de Salud Carlos III, Madrid, Spain; Department of Medicine, School of Medicine and Health Sciences, University of Barcelona, Spain; Institute of Neurosciences (UBNeuro), University of Barcelona, Spain; and Barcelona Clinic Schizophrenia Unit, Institut d'Investigacions Biomèdiques August Pi i Sunyer (IDIBAPS), Barcelona, Spain; Digital Innovation Group, Bipolar and Depressive Disorders Unit, Institute of Neuroscience, Department of Psychiatry and Psychology, Hospital Clínic de Barcelona, Spain; Department of Psychiatry and Psychology, Institut d'Investigacions Biomèdiques August Pi i Sunyer (IDIBAPS), Barcelona, Spain; Department of Medicine, School of Medicine and Health Sciences, University of Barcelona, Spain; Institute of Neurosciences (UBNeuro), University of Barcelona, Spain; and Barcelona Clinic Schizophrenia Unit, Institut d'Investigacions Biomèdiques August Pi i Sunyer (IDIBAPS), Barcelona, Spain; Institut d'Investigacions Biomèdiques August Pi i Sunyer (IDIBAPS), Barcelona, Spain; Department of Medicine, School of Medicine and Health Sciences, University of Barcelona, Spain; Institute of Neurosciences (UBNeuro), University of Barcelona, Spain; and Imaging of Mood- and Anxiety-Related Disorders (IMARD) Group, Institut d'Investigacions Biomèdiques August Pi i Sunyer (IDIBAPS), Barcelona, Spain; Institut d'Investigacions Biomèdiques August Pi i Sunyer (IDIBAPS), Barcelona, Spain; Department of Medicine, School of Medicine and Health Sciences, University of Barcelona, Spain; Institute of Neurosciences (UBNeuro), University of Barcelona, Spain; Imaging of Mood- and Anxiety-Related Disorders (IMARD) Group, Institut d'Investigacions Biomèdiques August Pi i Sunyer (IDIBAPS), Barcelona, Spain; Early Psychosis: Interventions & Clinical Detection (EPIC) Laboratory, Department of Psychosis Studies, Institute of Psychiatry, Psychology & Neuroscience, King's College London, UK; and Center for Psychiatry Research, Department of Clinical Neuroscience, Karolinska Institutet, Sweden; Institut Pascal (UMR 6602), Department of Psychiatry, CHU Clermont-Ferrand, University of Clermont Auvergne, CNRS, Clermont Auvergne INP, France; and Association Française de Psychiatrie Biologique et Neuropsychopharmacologie (AFPBN), Saint Germain en Laye, France; Centre for Affective Disorders, Institute of Psychiatry, Psychology & Neuroscience, King's College London, UK

**Keywords:** Bipolar disorder, mania, depression, physiological data, digital biomarkers

## Abstract

**Background:**

Bipolar disorder is highly prevalent and consists of biphasic recurrent mood episodes of mania and depression, which translate into altered mood, sleep and activity alongside their physiological expressions.

**Aims:**

The IdenTifying dIgital bioMarkers of illnEss activity and treatment response in BipolAr diSordEr with a novel wearable device (TIMEBASE) project aims to identify digital biomarkers of illness activity and treatment response in bipolar disorder.

**Method:**

We designed a longitudinal observational study including 84 individuals. Group A comprises people with acute episode of mania (*n* = 12), depression (*n* = 12 with bipolar disorder and *n* = 12 with major depressive disorder (MDD)) and bipolar disorder with mixed features (*n* = 12). Physiological data will be recorded during 48 h with a research-grade wearable (Empatica E4) across four consecutive time points (acute, response, remission and episode recovery). Group B comprises 12 people with euthymic bipolar disorder and 12 with MDD, and group C comprises 12 healthy controls who will be recorded cross-sectionally. Psychopathological symptoms, disease severity, functioning and physical activity will be assessed with standardised psychometric scales. Physiological data will include acceleration, temperature, blood volume pulse, heart rate and electrodermal activity. Machine learning models will be developed to link physiological data to illness activity and treatment response. Generalisation performance will be tested in data from unseen patients.

**Results:**

Recruitment is ongoing.

**Conclusions:**

This project should contribute to understanding the pathophysiology of affective disorders. The potential digital biomarkers of illness activity and treatment response in bipolar disorder could be implemented in a real-world clinical setting for clinical monitoring and identification of prodromal symptoms. This would allow early intervention and prevention of affective relapses, as well as personalisation of treatment.

Bipolar disorder is a severe, relapsing-remitting mental illness that affects approximately 1% of the world's population regardless of nationality, ethnic origin or socioeconomic status, and is ranked as the 17th leading source of disability worldwide.^1^ Individuals with bipolar disorder present biphasic recurrent mood episodes of mania, characterised by a reduced need for sleep, increased activity, expansive mood and behaviour; and depression, characterised by decreased activity and energy, sadness, and social withdrawal.^2^ Mood recurrences play a significant role in bipolar disorder prognosis, as well as the long-term subthreshold mood symptoms that have a negative impact on patients’ quality of life, cognition and life expectancy.^3^ Indeed, suicide and all-cause mortality rates are increased in individuals with bipolar disorder.^4^

Despite the pharmacological treatment guidelines for acute episodes or maintenance phases of bipolar disorder being well established, and treatments being highly effective,^5,6^ drug choice is primarily dictated by trial and error, based on specific clinical phenotypes of patients. Thus, around 30–55% of individuals with bipolar disorder selected for a specific treatment will show a poor response, so that clinical phenotypes do not have sufficient predictive validity on their own.^7^ The variability in response to pharmacological treatments in bipolar disorder is poorly understood, and response cannot be predicted at the outset, but requires a lengthy treatment trial. During these uncertain trial periods, adverse outcomes are frequent, such as side-effects, tolerability and clinical adverse consequences including mood switches, mood relapses and even suicide attempts.^8^

Hence, biomarkers capable of identifying illness activity and predicting response to pharmacological treatments will enable a better definition of criteria for patient stratification to establish timely/early and personalised treatments.^9^ This would improve short- and long-term management and prognosis of bipolar disorder, and is likely to reduce the risk of suicidal behaviours.^10^ Despite some advances in genetics, peripheral blood, neurophysiological biomarkers and neuroimaging correlates, increasing evidence suggests the presence of multiple biases within the existing body of scientific literature on biomarkers related to the most debilitating mental disorders, including bipolar disorder.^11^ As matter of fact, contrary to initial optimism, none of the proposed biomarkers have reached or are going to reach real-world clinical practice in the foreseeable future.^12^

In the past decade, alongside the mentioned research endeavours, advancements in miniaturisation and internet connectivity of sophisticated digital devices have facilitated their extensive availability and adoption across various daily activities among people of diverse backgrounds worldwide. Wearable technologies, such as smartwatches, smart bands and actigraphs, are worn throughout the day and are capable of impartially recording intricate details about users’ behaviours, sleep cycles and cognitive patterns in real time – a concept referred to as digital phenotyping.^12,13^

Bipolar disorder serves as an optimal diagnostic framework for digital phenotyping in mental health because of its characteristic biphasic nature, particularly evident during acute mood episodes, which manifest as altered mood, sleep and activity, and accompanying physiological expressions. Recent advancements in research-grade wearable technologies enable the capture of these alterations. Integrating these digital physiological signals with illness activity and treatment response holds the potential to identify digital biomarkers.^14^ For instance, wearable devices collecting actigraphy data can detect disrupted sleep patterns in individuals with remitted bipolar disorder,^15^ as well as depressive symptoms.^16^ Furthermore, wearable devices equipped with sensors for blood volume pulse have demonstrated variations in heart rate variability (HRV) between individuals with bipolar disorder and healthy controls,^17^ as well as in different affective states within bipolar disorder.^18^ Moreover, individuals with bipolar and unipolar depression and suicidal tendencies have exhibited autonomic dysfunction, which can now be monitored through hypo-reactive electrodermal activity (EDA).^19^ Traditionally, capturing EDA required expensive and intricate laboratory equipment until just a few years ago. However, recent advancements have led to the integration of sensors in research-grade wearable devices, enabling the continuous collection of EDA data.^20^ Despite these encouraging findings, the specific roles of these digital signals and their longitudinal potential in measuring illness activity and treatment response in bipolar disorder remain inadequately explored.^21,23^

The convergence of advancements in machine learning with the enhanced precision of state-of-the-art, research-grade wearable devices offers the potential to discern physiological patterns related to illness activity and treatment response in bipolar disorder. With this in mind, we introduce the rationale, research questions and protocol of the IdenTifying dIgital bioMarkers of illnEss activity and treatment response in BipolAr diSordEr with a novel wearable device (TIMEBASE) project. The primary objective of TIMEBASE is to identify digital biomarkers associated with illness activity and treatment response in bipolar disorder.

## Method

### Study design and objectives

We have designed a longitudinal, observational, exploratory single-centre study with a fully pragmatic design integrated into existing real-world clinical practice, providing minimal disruption both for clinicians and patients. The research questions to be answered by the TIMEBASE project are stated in Table 1.

**Table 1:** Research questions for the TIMEBASE study, in sequential order

Primary	Are there specific patterns of physiological data according to the **severity** of an acute affective episode (manic, depressive, and/or mixed) at the intra-individual level?
	Are there specific patterns of physiological data between patients at different acute **affective phases** (manic, depressive, mixed and euthymia), and compared with healthy controls? Are there differences between acute bipolar from unipolar depression?
	Can physiological data predict **treatment response** in affective episodes?
Secondary	Are there specific patterns of physiological data associated with affective **symptoms**?
	If present, **which digital biomarkers** are explaining intra- and between-individual differences, and which are associated with each symptom?
	If present, can the former digital biomarkers **generalise** across patients?
	Does the **severity of symptoms** influence the accuracy of the former associations?
	When considering **sleep and wake** times separately, which is more useful for the former associations? Do digital biomarkers vary between sleep and wake times?
	Do potential **confounders** influence digital biomarkers and the former associations? pharmacological treatments (type and doses) comorbid medical conditions physical activity atmospheric (weather, humidity, temperature) diet (caffeine, water intake)
	How do participants **cluster** according to physiological data?
Future research questions	If digital biomarkers of illness activity and treatment response are identified by this project, the next research questions would be: Are there specific patterns of physiological data for **prodromal signs** of manic and depressive episodes? Could a wearable device alert patients and potentially prevent an affective episode?

TIMEBASE, IdenTifying dIgital bioMarkers of illnEss activity and treatment response in BipolAr diSordEr with a novel wearable device.

#### Hypotheses

We hypothesise that physiological data captured through a wearable device will accurately and objectively (a) reflect illness activity during acute affective episodes in bipolar disorder, (b) differentiate between different acute affective episodes and (c) predict treatment response.

#### Objectives

The primary objective of the study is to identify digital signatures of illness activity in bipolar disorder at the intra- (acute, response, remission) and between-individual (manic, depressed, euthymic, healthy controls) levels, and identify digital signatures of treatment response.

The secondary objectives are to (a) correlate physiological data to specific affective symptoms; (b) identify which digital biomarkers are most relevant for the former models; (c) assess generalisation across patients, to evaluate the role of the severity of symptoms, circadian rhythms and confounders in the identification of illness activity and treatment response; and (d) assess clustering among participants.

### Sample and study design

**Fig. 1:**
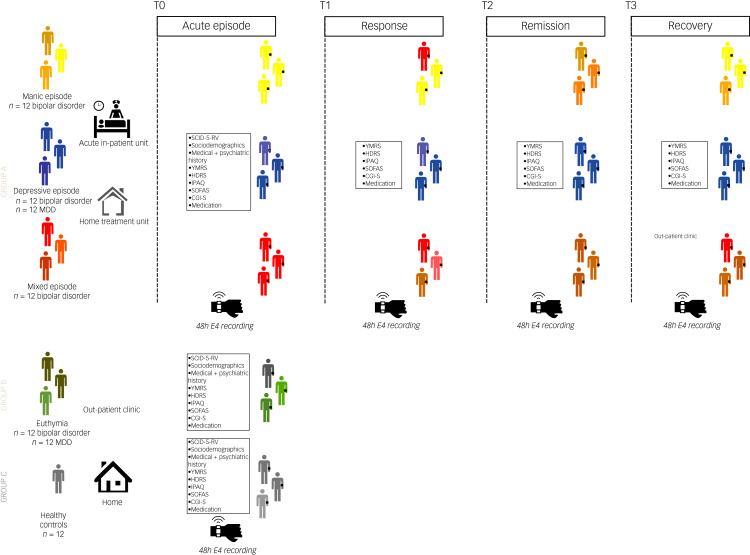
Study design. Group A: patients with acute affective episodes. A consecutive group of 48 individuals: A1: 12 manic episodes in bipolar disorder. A2: 24 major depressive episodes, of which 12 were bipolar disorder and 12 were MDD. A3: 12 mixed features manic episodes in bipolar disorder. Patients will be monitored with a wearable device at four time points: T0, acute affective episodes (manic, depressive, and mixed) at the beginning of hospital admission; T1, response of the episode, usually at mid-admission; T2, remission of the episode, usually at the end of admission or shortly after discharge; and T3, recovery (2 months after sustained remission), at out-patient setting after discharge. Recordings will be according to the psychopathological status of patients (acute, response, remission, recovery), so that the proposed timings during admission (beginning, mid-admission, end of admission, after discharge) are oriented according to usual clinical experience. Group B: patients with euthymia, an independent group of 24 consecutive patients (12 bipolar disorder and 12 MDD). They will be monitored at the out-patient clinic. Group C: 12 healthy controls will be monitored during their usual daily routine. CGI-S, Clinical Global Impression Scale: Severity Of Illness; HDRS, Hamilton Depression Rating Scale total score; IPAQ, Short Scale International Physical Activity Questionnaire; MDD, major depressive disorder; PANSS, Positive And Negative Syndrome Scale; SCID-5-RV, Structured Clinical Interview for DSM-5, Research Version;SOFAS, Social and Occupational Functioning Assessment Scale; YMRS, Young Mania Rating Scale total score.

The study will include a total of 84 individuals from three independent groups (Fig. 1). Potential participants will be identified by their clinicians, namely psychiatrists, at out-patient clinics, acute in-patient units or home hospitalisation settings. Healthy controls will be recruited from a convenient selection of researchers and family members.

#### Inclusion criteria

The inclusion criteria for group A are as follows: (a) age 18–75 years; (b) current acute manic episode (A1), major depressive episode (A2) or mixed features manic episode (A3) in the context of bipolar disorder (for groups A1, A2 and A3) and major depressive disorder (MDD) (for 12 patients from group A2), according to the DSM-5 criteria^24^ and confirmed by a semi-structured interview using the Structured Clinical Interview for DSM-5, Research Version (SCID-5-RV)^25^; (c) requiring admission to the acute in-patient or home hospitalisation units; and (d) willing and able to give consent (reconfirmed on clinical remission).

Current acute DSM-5 affective episodes will be measured at time point 0. Symptom response will be measured at time point 1, defined as a 30% improvement in the Young Mania Rating Scale (YMRS)^26^ score or 17-item Hamilton Depression Rating Scale (HDRS)^27^ score. Time point 2 will measure symptomatic remission, defined as a YMRS or HDRS score ≤7. Time point 3 will assess remission, defined as ≥8 weeks of sustained remission. If patients do not show clinical improvement (i.e. 30% improvement in clinical scores after time point 0), such as treatment-resistant patients, they will only be recorded at time point 0 (acute phase). Likewise, patients attaining clinical remission (i.e. YMRS or HDRS ≤7) rapidly (e.g. in <1 week), will skip the time point 1 recording and will be recorded at time point 0 (acute) and time point 2 (remission phase). Patients in acute phases will be assessed by the research team on a weekly basis to assess psychopathological changes corresponding to response or remission.

The inclusion criteria for group B are as follows: (a) age 18–75 years; (b) patients with a current diagnosis of bipolar disorder (*n* = 12) or MDD (*n* = 12), according to DSM-5 criteria and confirmed with a semi-structured interview (SCID-5-RV); (c) sustained YMRS and HDRS scores of ≤7 for at least 8 weeks; and (d) willing and able to give consent.

The inclusion criteria for group C are as follows: (a) age 18–75 years; (b) no current or previous psychiatric disorder, according to the DSM-5 criteria and confirmed with a semi-structured interview (SCID-5-RV), excluding nicotine substance use disorder; and (c) willing and able to give consent.

#### Exclusion criteria for all groups

The exclusion criteria for all groups are as follows: (a) presence of severe cardiovascular or neurological medical conditions that may lead to autonomic dysfunction, ongoing cardiovascular arrhythmia or pacemaker use; (b) current comorbid substance use disorder as per DSM-5 criteria, excluding nicotine substance use disorder; (c) current comorbid psychiatric disorder causing significant interference with symptoms; (d) current pharmacological treatment involving beta-blockers or other medications affecting the autonomic nervous system; and (e) ongoing pregnancy.

#### Sample size

Determining the required number of participants is contingent on the primary end-point, which is the identification of digital signatures indicating illness activity in bipolar disorder. There is a lack of precedent and similar studies reporting α-error, 1–β error and a mean/s.d. ratio assessing severity in bipolar disorder from digital biomarkers to calculate the sample size. Given the exploratory nature of this study because of the totally novel measuring methods employed, we adopted a conservative pragmatic approach to estimate the sample size with balanced and practical feasible groups, as recommended in pilot studies.^28^ Indeed, previous works using wearable physiological data to identify digital signatures of illness activity in bipolar disorder find significant differences with samples smaller than 20 per group.^29,30^ Our approach seeks to maximise the sample size according to our recruitment capacities for the period of the study and the availability of patients with acute affective episodes at our centre. Thus, taking into account the multiple and continuous data points for each individual during the study period provided by the wearable devices, we have determined that a minimum of 12 patients each for acute affective episode (manic, depressive and mixed), 12 euthymic patients and 12 healthy controls will be necessary to establish composite models to identify illness activity patterns. Twenty-four patients with MDD (12 acute and 12 euthymic) will be added to compare with bipolar depression and euthymia. Of note, from a total of 84 participants, 48 will be recorded longitudinally at different time points, according to the resolution of an acute episode. Therefore, although the sample size is limited, it will have the strength of being prospective and allowing for intra-individual comparisons. The sample size determined for the initial phase of the study could pose a challenge for machine learning because of the constraints on sample sizes for both training and testing data-sets. Consequently, the findings from our analyses will be validated using larger sample sizes in subsequent phases of the study.

### Assessments

#### Sociodemographic and clinical assessment

At baseline (time point 0), the following sociodemographic and clinical data will be gathered: age, gender, psychiatric diagnoses according to DSM-5 criteria, medical and psychiatric comorbidities, duration of illness, frequency of past manic and depressive episodes, longitudinal course specifiers (such as predominant polarity, seasonality and rapid cycling), history of mental illness among first-degree relatives and prior substance misuse patterns.

#### Symptoms, severity and functional assessment

Psychopathological assessments will be conducted with the YMRS for manic symptoms, the 17-item HDRS for depressive symptoms and the Positive and Negative Syndrome Scale (PANSS)^27^ for psychotic symptoms. Disease severity will be assessed with the Clinical Global Impression Scale: Severity of Illness (CGI-S)^31^, with higher scores indicating greater severity. The Social and Occupational Functioning Assessment Scale (SOFAS)^32^ will be administered for functional evaluation without the influence of symptom severity. It employs a numerical scale ranging from 1 to 100, where higher scores signify improved functionality. Moreover, the Stroop Color Word Test (CWT)^33^ will be performed as a stress-elicitation task for the study of autonomic nervous system alterations in mood episodes, on the basis of psychological, physiological and biochemical responses. Clinical assessments will be performed cross-sectionally for groups B and C and at different time points (time point 0: acute, time point 1: response, time point 2: remission, time point 3: recovery) for group A (Fig. 1).

#### Physical activity assessment

Physical activity will be evaluated using the Short Scale International Physical Activity Questionnaire (IPAQ), which assesses various types of activity such as walking, moderate-intensity activities and vigorous-intensity activities. The assessment includes collecting data on the frequency (measured in days per week) and duration (time per day) of each specific type of activity. The continuous score IPAQ results will be expressed in metabolic equivalent of task per min (MET-min) per week. This score is calculated by multiplying the MET assigned to each activity (8 MET-min for vigorous, 4 MET-min for moderate and 3.3 MET-min for walking) by the number of days it was performed during a week. MET-min corresponds to oxygen consumption during rest, and is equivalent to 3.5 ml of oxygen per kg of body mass per min. Physical activity assessments will be conducted cross-sectionally for groups B and C, and at various time points (time point 0: acute, time point 1: response, time point 2: remission, time point 3: recovery) for group A (Fig. 1). Indeed, objective wearable-collected actigraphy measures will be correlated with subjective physical activity assessments (IPAQ), to validate the objective measurements.

#### Pharmacological treatments

All pharmacological treatments (both psychopharmacological and others) as described in international treatment guidelines will be included registering their generic name, starting day and dose per day in each of the 48-h registers: cross-sectionally for groups B and C, and at different time points (time point 0: acute, time point 1: response, time point 2: remission, time point 3: recovery) for group A (Fig. 1). Because of the pragmatic design, treatment decisions (including inpatient discharge) will be exclusively determined by the judgement of the clinicians in charge of the case and patients’ agreement resembling routine clinical care. The researchers performing the recruitment of the study will not be involved in any clinical decisions of the patients included.

#### Other potential confounders

Other potential confounders, such as atmospheric conditions (humidity, temperature), diet (caffeine, water intake) and quantitative tobacco use, will be registered during each recording.

### Registration of physiological data

During each assessment – cross-sectionally for groups B and C, and at different time-points (time point 0: acute, time point 1: response, time point 2: remission, time point 3: recovery) for group A (Fig. 1) – patients and healthy controls from groups B and C will be provided with an E4 Empatica wristband,^34^ which they will wear for approximately 48 h, limited by the device's battery life. This non-interventional study ensures that individuals’ behaviour remains unaltered apart from the requirement to wear the wristband. Patients from group A admitted to the psychiatric in-patient unit are required to remain in the hospital until discharge, adhering to standard practice for acute patients. During in-patient admission, patients follow a structured routine, including specific meal times (e.g. breakfast at 08:30 h) and sleep schedules (22:30 h to 00:30 h), as well as engaging in daily activities such as consultations with psychiatrists and therapy groups. Patients may nap during the day if necessary. Given the relatively uniform conditions during in-patient admissions, recordings at time points 0–2 are typically conducted in this setting, reducing variability between individuals. However, patients from group A admitted to the home hospitalisation units or out-patient setting (a minority of cases) are not subject to mobility restrictions. In all cases, participants are instructed to wear the wristband during their daily activities without altering their behaviour. They are also instructed to put on the wristband themselves at the start of recording, and researchers will ensure adequate sensor contact with the wrist surface. Participants are advised to remove the device when showering, to maintain its integrity.

Empatica E4 devices are equipped with sensors that gather physiological data at varying sampling rates. During each recording session, the physiological data signals are obtained in either raw or processed formats. The raw data includes measurements from the following channels: three-dimensional acceleration in space over time, on *x-*, *y*- and *z*-axes ((ACC) sampled at 32 Hz); EDA (sampled at 4 Hz); skin temperature ((TEMP) sampled at 4 Hz) and blood volume pulse ((BVP), sampled at 64 Hz). The processed format includes inter-beat intervals (IBI), which represent the time between two consecutive heart ventricular contractions, and heart rate, sampled at 1 Hz. The BVP signal is captured using a photoplethysmography sensor, which measures volume changes in the blood. It is worth noting that Empatica directly provides IBI as part of its output, and calculates it by detecting peaks (beats) of the BVP and determining the lengths of the intervals between adjacent beats. Similarly, heart rate is computed from IBI by using a proprietary algorithm. Both algorithms are optimised to filter out beats containing artifacts.^34,35^

There are many research-grade wearable devices available. For this project, we took into account the following factors when choosing the Empatica E4: (a) signals of interest to be captured (e.g. actigraphy, EDA, HRV, temperature) and tasks performed (e.g. stress-elicitation); (b) data availability, as some wearable devices only share general data (e.g. mean sleep time) and not fine-grained raw data, so that visual inspection, quality control of data and specific detailed analyses cannot be performed; (c) study participants (bipolar disorder, MDD, healthy controls); (d) study setting (in-patients, out-patients); (e) confidentiality of data and (f) previous literature supporting the device. Indeed, the Empatica E4 wearable device was selected for this study because of all of the aforementioned reasons. It is capable of measuring all signals of interest (especially HRV and EDA) and it provides raw fine-grained data, thus allowing to process data according to the needs of the study. Moreover, it is a device without direct internet connection and shows resistance to different physical situations, thus preserving confidentiality in patients experiencing acute affective episodes (who may lack insight and present with behavioural alterations). Certainly, the in-patients involved are in a tightly controlled environment that limits the use of the device's communication with the external environment. Finally, previous literature in bipolar disorder was already present supporting the use of this same device. Limited battery life could be considered a limitation if the objective of the study was to capture day-to-day variations in physiological signals. However, acute affective episodes show day-to-day fluctuations and response and recovery can only be assessed after symptoms have been reduced in a sustainable way over the course of several days or weeks. This fact, and the need for sequentially evaluating the psychopathological status, suggested that 48-h registers would adequately capture mood changes according to the needs of the study.

### Statistical analyses

The physiological data provided by the wearable device (channels: ACC, EDA, TEMP, BVP, IBI, HR) are provided at different sampling rates (from 64 to 1 Hz) and contain artifacts that may alter their validity.^34^ Thus, pre-processing methods to remove invalid data (artifacts),^36^ as well as time-alignment methods, will be implemented. After pre-processing the data, to identify digital signatures of illness activity, recorded sessions will be divided into train, validation and test sets, and different machine learning models will be applied and their accuracies compared. Feature importance analysis will be used to identify which digital biomarkers explain the former models. Out-of-sample generalisation performance of the former models will be tested in physiological data from unseen patients (i.e. the physiological data from approximately 80% of patients will be used to build the models, and 20% will be used for model generalisation). Once possible digital signatures of illness activity have been accurately determined, treatment response will be assessed using the same methods. Finally, remote monitoring will be implemented with real-time identification of prodromal symptoms and early-intervention strategies.

### Ethics and confidentiality

The authors affirm that all procedures conducted in this study adhere to the ethical standards outlined by national and institutional committees on human experimentation and comply with the principles of the Helsinki Declaration of 1975, as revised in 2008. Approval for all procedures involving human patients was obtained from the Hospital Clinic Ethics and Research Board (approval numbers HCB/2021/104 and HCB/2021/1127). Before participation in the study, all participants will provide written informed consent. Data collection will be anonymous, and all data will be securely stored in encrypted servers in compliance with the General Data Protection Regulation and Health Insurance Portability and Accountability Act regulations.

## Results

Recruitment is ongoing.

## Discussion

Despite extensive biomarker research in mental disorders, there is a lack of valid biomarkers as supporting clinical tools in mental health compared with other areas of healthcare.^11^ Biomarkers may help to understand the pathophysiological basis of mental disorders, and thus have the potential to aid in precision medicine in the real world.^37^ The improved precision to continuously, ecologically, and massively collect physiological data, and the enhanced capacity to process and analyse them, hold great potential for the mental health field. Digital biomarkers could add value to the current biomarker research in mental disorders, and especially in bipolar disorder, because of its particular biphasic nature. The TIMEBASE project aims to explore digital biomarkers of illness activity and treatment response in bipolar disorder.

Previous works using wearable physiological data focused on differentiating acute versus remission states as a mere dichotomous classification problem.^29,30,38,41^ In contrast, our objective is to discern the severity of depression and mania (illness activity) in a dynamic and longitudinal manner, tracking the progression of mood episodes (time point 0: acute, time point 1: response, time point 2: remission, time point 3: recovery). We posit that quantifying the severity of affective episodes presents a more challenging yet clinically significant endeavour, offering the potential for a deeper and more thorough understanding of the condition.

Indeed, the design of the study combining longitudinal and cross-sectional monitoring of patients on acute affective episodes is expected to respond different but complementary research questions, using two analytical approaches: (a) On the one hand, cross-sectional analyses between different individuals during distinct mood states (including mania, depression, mixed states and euthymia) will determine whether there are specific patterns of physiological data between patients at different acute affective phases (research question 2); (b) on the other hand, longitudinal analyses within the same individual during the resolution of an affective episode (acute, response, remission and recovery) will allow us to determine whether there are specific patterns of physiological data according to the severity of an acute affective episode (research question 1), and whether physiological data can predict treatment response (research question 3).

In addition, it should be noted that in intra-individual comparisons (when comparing longitudinally within the same patient), heterogeneity resulting from individual characteristics (e.g. gait, gender, age, etc.) is partially controlled for (the patient is their own control). Therefore, intra-individual models should be more likely to capture signals of illness activity (physiological changes may be more related to psychopathology rather than simply to individual characteristics). In contrast, between-individual (cross-sectional) comparisons do not control for individual heterogeneity and illness activity signals are more likely to be distorted by external factors (e.g. humidity, atmospheric temperature or exercise), which will be registered and controlled for in the analyses.

Regarding specific symptoms of mania and depression, only two studies attempted to infer depression total scores based on wearable physiological data.^42,43^ Patients with the same total score on the YMRS and HDRS might have very different clinical presentations, because of to the multiple combinations of possible symptoms.^44,45^ For instance, one patient may display psychomotor agitation whereas the other may display psychomotor retardation (two items from HDRS), and yet both could score the same on the total HDRS. Therefore, when reducing a psychometric scale to its total score, highly relevant information for treatment personalisation is lost. In this project, we aim to overcome this limitation by trying to infer specific manic and depressive symptoms by using wearable physiological data.

Furthermore, generalisation of machine learning models on unseen patients is key to ensure that those can be used on a real-world clinical setting (i.e. applicable to different patients than those used to develop the model). To the best of our knowledge, previous studies did not attempt to assess generalisation using samples from participants other than those utilised for model development (i.e. they solely employed physiological data from identical participants both for training and testing phases).^29,30,38^ In contrast, our objective is to conduct model development and evaluation using data from different patients to evaluate out-of-sample generalisation. This task may be challenging, as the diversity among individuals and non-disease-related factors (e.g. gender, age, physical fitness) could affect physiological signals. Therefore, we will focus on machine learning systems that capture disease-relevant traits that are generalisable across patients, rather than those that overfit patient-specific patterns, and we will assess their performance accordingly.

Finally, previous works using wearable physiological data in patients with bipolar disorder have used particular digital signals like actigraphy,^29^ or combinations like actigraphy and EDA,^38^ but disregarded other parameters arbitrarily such as skin temperature or heart rate. Our objective is to incorporate physiological digital data from all channels provided by the E4 Empatica wearable device (including EDA, acceleration, skin temperature, blood volume pulse and heart rate),^34^ and evaluate the relevance of each channel in predictive models. This approach allows machine learning models to determine which physiological signal is most pertinent for each specific task. Furthermore, we will explore the role of smartphone-based data, including ecological momentary assessments,^46,48^ in identifying illness activity and treatment response during this project.

### Limitations

The study has limitations inherent to all observational studies and a novel method capturing a combination of physiological data that has not been extensively tested in bipolar disorder. The limited sample will be fully compensated with a method capable of capturing an unprecedented quantity of highly detailed, granular physiological data per individual, which will allow an accurate building of predictive models about illness activity and treatment response. Treatment variability will be mitigated by a common and uniform standard of acute episodes treatment at both the acute in-patient and home hospitalisation units. There is also the risk of loss to follow-up and the participant insufficiently or inadequately wearing the device, rendering the collected data useless. However, the recording sessions are relatively brief (around 48 h) and under close clinical care provided at the in-patient and home hospitalisation units treatment programme in the case of group A, which is potentially the most complex group. Moreover, invalid physiological data registered by the wearable device can be easily removed during the pre-processing phase. Selection bias will be mitigated by establishing an age- and gender-matched consecutive sample recruitment and reassessment by an independent researcher, with the unique exception of pharmacological treatments or medical conditions that might affect physiological data parameters. As a strength of the study, its prospective characteristics will allow us to control variables and device parameters with treatment doses, thus allowing to establish causal effect about multiple factors and outcomes. Wearable devices are an unobtrusive objective method of capturing data under real-world conditions, thus minimising a potential Hawthorne effect. In addition, these new monitoring methods allow for an intensive and detailed collection of physiological data compared with classical clinical research methods, in which subjective assessments are conducted in sparse clinical interviews. The large amount of fine-grained longitudinal information for each individual can allow us to investigate hypotheses that have been unattainable so far, as well as develop highly personalised models for a single individual, using small sample sizes.

Despite the outlined limitations, this project represents an innovative endeavour aimed at identifying digital biomarkers of illness activity and treatment response in bipolar disorder. However, the scarcity of precedent studies utilising these devices in bipolar disorder restricts its immediate validation against comparable data-sets. Similarly, the absence of similar methodologies in the literature and publicly available codes for pre-processing and analysis using machine learning necessitates their development from scratch, hindering the ability to compare our findings with others. Nevertheless, the outcomes of this project hold promise for advancing our understanding of the pathophysiology of bipolar disorder and affective disorders. The potential digital biomarkers of illness activity and treatment response identified in bipolar disorder could be integrated into real-world clinical settings for clinical monitoring and early identification of prodromal symptoms. This could facilitate timely intervention and prevention of affective relapses, as well as personalised treatment approaches for patients with bipolar disorder.

## Data Availability

The methodology and data that support the findings of this study will be available from the corresponding author, E.V., upon reasonable request.
